# ImmunoPET provides a novel way to visualize the CD103^+^ tissue-resident memory T cell to predict the response of immune checkpoint inhibitors

**DOI:** 10.1186/s13550-023-01062-6

**Published:** 2024-01-05

**Authors:** Xiaoyu Fan, Hans W. Nijman, Marco de Bruyn, Philip H. Elsinga

**Affiliations:** 1 Department of Nuclear Medicine and Molecular Imaging, University of Groningen, University Medical Center Groningen, Groningen, The Netherlands; 2 Department of Obstetrics and Gynecology, University of Groningen, University Medical Center Groningen, Groningen, The Netherlands

**Keywords:** CD103, Biomarkers, PET, T_RM_

## Abstract

**Background:**

Immune checkpoint inhibitors (ICIs) have made significant progress in oncotherapy improving survival of patients. However, the benefits are limited to only a small subgroup of patients who could achieve durable responses. Early prediction of response may enable treatment optimization and patient stratification. Therefore, developing appropriate biomarkers is critical to monitoring efficacy and assessing patient response to ICIs.

**Main body:**

Herein, we first introduce a new potential biomarker, CD103, expressed on tissue-resident memory T cells, and discuss the potential application of CD103 PET imaging in predicting immune checkpoint inhibitor treatment. In addition, we describe the current targets of ImmunoPET and compare these targets with CD103. To assess the benefit of PET imaging, a comparative analysis between ImmunoPET and other imaging techniques commonly employed for tumor diagnosis was performed. Additionally, we compare ImmunoPET and immunohistochemistry (IHC), a widely utilized clinical method for biomarker identification with respect to visualizing the immune targets.

**Conclusion:**

CD103 ImmunoPET is a promising method for determining tumor-infiltrating lymphocytes (TILs) load and response to ICIs, thereby addressing the lack of reliable biomarkers in cancer immunotherapy. Compared to general T cell markers, CD103 is a specific marker for tissue-resident memory T cells, which number increases during successful ICI therapy. ImmunoPET offers noninvasive, dynamic imaging of specific markers, complemented by detailed molecular information from immunohistochemistry (IHC). Radiomics can extract quantitative features from traditional imaging methods, while near-infrared fluorescence (NIRF) imaging aids tumor detection during surgery. In the era of precision medicine, combining such methods will offer a more comprehensive approach to cancer diagnosis and treatment.

## Background

Cancer immunotherapy has changed the treatment strategy across multiple types of tumors, introducing a new era in cancer treatment. Cancer immunotherapy is based on activating and supporting the immune system of the body to recognize and kill tumor cells. The antitumor immune response is enhanced and prolonged by sustained recognition of tumor antigens. Subsequently, specific cytotoxic T cells differentiate into natural memory T cells, providing long-term immune memory protection even without primary antigen stimulation [1]. Thus, immunotherapy is more likely to achieve long-term survival than conventional therapy.

A representative example of cancer immunotherapy is the use of immune checkpoint inhibitors [2, 3]. Immune checkpoint molecules, such as programmed cell death protein (PD-1), programmed cell death ligand 1 (PD-L1), and cytotoxic T-lymphocyte-associated antigen 4 (CTLA-4), are immune system regulators that maintain self-tolerance in the immune system and prevent immune responses from damaging tissue. However, high expression of PD-L1 in tumor cells and other cell types in the tumor microenvironment leads to engagement of PD-1, resulting in the suppression of T cell growth, survival, and other effector functions. Due to their immunosuppressive regulatory properties, inhibitory checkpoint molecules have become prominent targets during the therapeutic development process for cancer immunotherapy [4].

**Fig. 1:**
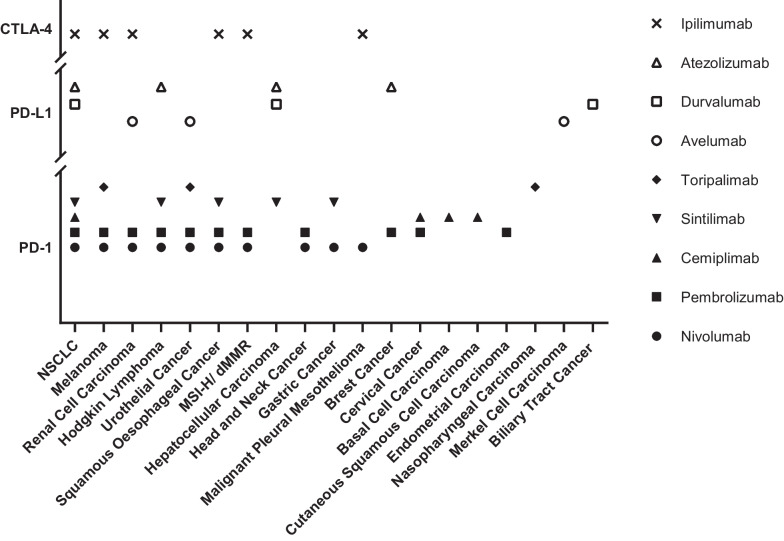
Indications for current marketed immune checkpoint inhibitors, data collected from the EMA and NMPA websites until June 6th, 2023. NSCLC: Non-Small Cell Lung Cancer, MSI-H: Microsatellite Instability-high, dMMR: Mismatch repair deficient

The number of clinical trials related to checkpoints inhibitors has rapidly increased over the past decade [5]. Since ipilimumab was approved as the first immune checkpoint inhibitor for the treatment of several solid tumors, various immune checkpoint inhibitors have been developed and entered the market (Fig. 1). Immune checkpoint inhibitors are widely used in treating non-small cell lung cancer and other cancers for which the therapy is often approved, including melanoma, renal cell carcinoma, Hodgkin lymphoma, and urothelial cancer.

However, the objective response rate (ORR) utilizing immune checkpoint inhibitors alone is only 10% to 30% in most unselected solid tumors. One exceptional case is classic Hodgkin’s lymphoma, with the ORR of more than 60% [6]. Although durable responses are achieved in some patients, most do not benefit. Thus, reliable biomarkers are needed to predict the patient’s response to immune checkpoint inhibitors. Such biomarkers could incorporated into the prognostic decision-making system to guide clinical immunotherapy applications.

The predictive value of biomarkers among effective immunotherapies, such as PD-L1 and tumor mutational burden (TMB), continues to be tested in several clinical trials. From these studies, it became clear that the predictive value of these biomarkers is limited.

Programmed death ligand 1 (PD-L1) emerged as the first potential predictive biomarker for immune checkpoint inhibitors. However, a study of Andrew and Vaibhav [7] assessed PD-L1 as a predictive biomarker across all FDA-approved immune checkpoint inhibitors, analyzing 45 primary studies spanning from 2011 to April 2019; encompassing 15 different tumor types showed that PD-L1 was predictive in only 28.9% of all approvals and was either not predictive (53.3%) or not tested (17.8%) in the remaining cases. In conclusion, this study suggested that PD-L1 expression has limitations as a predictive biomarker. Moreover, a clinical study with combined nivolumab and ipilimumab or monotherapy in untreated melanoma among patients with PD-L1-negative tumors revealed moderate objective response rates of 41.3% in the nivolumab group, 54.8% in the nivolumab-plus-ipilimumab group, and 17.8% in the ipilimumab group, respectively [8].

As an emerging predictive biomarker for immune checkpoint inhibitors, the rationale of TMB is based on the hypothesis that a large number of mutations in the exonic region of somatic cells will lead to increased production of new antigens that can be recognized by CD8 + T cells and lead to the activation of T cell and antitumor immune responses. The therapeutic effects of immune checkpoint inhibitors also depend on the number of tumor-specific T cells, suggesting that TMB can be considered a potential immunotherapy biomarker [15, 16]. Although tumor mutational burden-high (TMB-H) was successful in predicting the outcome of ICIs in multiple cancer, recent clinical studies have shown that TMB-H failed to predict immune checkpoint inhibitors response in breast cancer, prostate cancer, and glioma [21, 22]. An obvious limitation of this technique will be that the optimal threshold for predicting response to immunotherapy may vary depending on tumor histology due to the enormous heterogeneity of different tumors [23]. In conclusion, a standard threshold for TMB as a potential biomarker for predicting response to immunotherapy may not be appropriate for all types of cancer.

Given the heterogeneity of the immune environment across different tumor types, finding predictive biomarkers has been challenging. Considering the shortage of effective biomarkers that could help to predict the clinical outcomes of immune checkpoint inhibitors, it is crucial to establish new measurement techniques for biomarkers to predict the response of cancer patients to immunotherapy. Herein, a new potential biomarker on tissue-resident memory T cells named CD103 is proposed, and the potential application of CD103 ImmunoPET to visualize the CD103 + tissue-resident memory T cell to predict the response of immune checkpoint inhibitors is also further discussed.

## Main text

### ImmunoPET imaging of tissue-resident memory T cell as a potential method for determining tumor-infiltrating lymphocytes (TILs) load and response to Immune checkpoint inhibitors

Tumor immunotherapy has unique advantages over traditional treatment modalities by harnessing the power of the body’s immune system to combat cancer cells. Due to this unique characteristics, biomarkers in the tumor microenvironment which indicate the immune response could potentially predict the patients’ response of Immune checkpoint inhibitors. Nowadays, such novel identified targets are often developed into ImmunoPET tracers which is a revolutionary molecular imaging modality that combines the outstanding targeting specificity of monoclonal antibodies (mAb) with the inherent sensitivity of PET.

### *CD103 ImmunoPET to predict the* response to immune checkpoint inhibitors

TILs refer to a group of white blood cells that leave the bloodstream and reside in the tumor microenvironment (TME) [9]. In the last three decades, studies have shown that high levels of TILs correlate with a favorable long-term prognosis in patients affected by various solid tumors such as metastatic melanoma [10], breast cancer [11], ovarian cancer [12], and metastatic colorectal cancer [13]. However, not every type of TIL plays the same crucial role in the anti-cancer immune response. One systematic review with a meta-analysis, which aims to establish pooled estimates for survival outcomes based on the presence of TILs in cancer, shows that CD3^+^ TILs had a positive effect on survival with a hazard ratio (HR) of 0.58 for death, as did CD8^+^ TILs with an HR of 0.71, however, FoxP3^+^ regulatory TILs were not linked to overall survival, with an HR of 1.19 [14]. As concluded, a specific TILs subset that can accurately forecast the efficacy of immune checkpoint inhibitors might be identified as a valuable biomarker.

CD103, also defined as Integrin alpha E (ITGAE), is a heterodimeric integrin membrane protein composed of an alpha chain and a beta chain, which is composed of Integrin alpha E (ITGAE) and Integrin beta 7 (β7–ITGB7)[15].

CD103 is expressed on multiple subsets of T cells and dendritic cells [16, 17]. The primary established function of CD103 in vivo is binding to E-cadherin and mediating the adhesion of intra-epithelial T-lymphocytes to epithelial cell monolayers. Tissue-resident memory T cells (T_RM_) are a subset of long-lived memory T cells characterized by their non-recirculating pattern of localization to non-lymphoid peripheral tissues. T_RM_ is crucial in defending the skin and non-lymphoid organs from bacterial and viral infections [18, 19]. A subtype of tumor-infiltrating T cells known as CD103^+^ resident-like tumor-infiltrating T cells has recently been identified in the tumor microenvironment and has been shown to be a predictive biomarker in solid cancers [20, 21].

As shown in Table 1, in the past five years, multiple clinical studies indicated that the high level of CD103^+^ TILs in tumors showed prognostic benefits across multiple types of solid cancer, including cervical cancer, head and neck squamous cell carcinoma, lung and bladder cancer, cholangiocarcinoma, gastric cancer, ovarian cancer, esophageal squamous cell carcinoma, colorectal cancer, and melanoma.

**Table 1:** Overview of the role of CD103 concerning prognostic benefit across multiple types of solid cancer

Tumor histology	Summary
Cervical cancer [22]	High infiltration of CD103^+^ T cells was associated with improved survival in the radio (chemo) therapy group
Head and neck squamous cell carcinoma [23]	Increases in the tumor-reactive CD103^+^ CD39^+^ CD8^+^ TIL coalbeds a potential biomarker of anti-OX40 clinical activity
Lung and bladder cancer [24]	The presence of CD103^+^ CD8^+^ T_RM_, quantified by tracking intra-tumoral CD103 expression, can predict treatment outcomes, suggesting that patients who respond to PD-1/PD-L1 blockade are those who exhibit an ongoing antitumor T cell response
Cholangiocarcinoma [25]	CD69^+^CD103^+^ T_RM_-like CD8^+^ TILs represent prominent tumor-specific immune responses and hold promise as a potential therapeutic target in ICC patients
Gastric cancer [26]	CD103^+^ T cells, accompanied by CD8^+^ T cells, were observed in the tumor epithelium and were associated with a better prognosis in gastric cancer. Furthermore, CD103^+^ T cells were located around tertiary lymphoid structure (TLS), and patients with high CD103 had richer TLS. Patients with CD103-high cells and TLS-rich tissues had a better prognosis than patients with CD103-low cells who were TLS-poor. Moreover, for patients who received PD-1 blockade therapy, CD103 levels were high and TLS-rich, predicting a potential response
Ovarian cancer [27]	CD103-positive tissue-resident memory-like CD8^+^ T cells (CD8^+^ CD103^+^ T_RM_) is associated with improved prognosis across malignancies, including high-grade serous ovarian cancer (HGSOC)
Esophageal squamous cell carcinoma [28]	CD103^+^ TILs play an essential role in the tumor microenvironment, and intra-tumoral CD103^+^ TILs could serve as a promising prognostic marker in ESCC
Colorectal cancer [29]	The density of tumor-infiltrating CD8^+^T cells or the number of resident CD103^+^ CD8^+^T cells in colorectal tissues could be a significant prognostic predictor for this malignancy
Melanoma [21]	CD103^+^ tumor-resident CD8^+^ T cells are associated with improved survival in immunotherapy naïve melanoma patients and expand significantly during anti-PD-1 treatment

Several potential mechanisms could explain the clinical benefits of CD103^+^ resident-like tumor-infiltrating T cells. When the CD103 binds to the epithelial cell marker E-cadherin, it helps the location and retention of T_RM_ in epithelial tumor regions. This interaction is also required for polarized exocytosis of lytic granules, which might lead to targeted tumor cell death. Furthermore, T_RM_ highly expressed granzyme B, IFN, and TNF, indicating their cytotoxic character. T_RM_ cells also predominantly express checkpoint receptors such as PD-1, CTLA-4, and Tim-3, providing the target for immune checkpoint inhibition therapy [20].

Mechanistic studies also indicate that CD103 is induced after specific activation of T cells against their cognate target [30–32], and the number of CD103^+^ cells increases significantly during successful immune checkpoint inhibitor treatment in lung and bladder cancer [24], melanoma [21], and non-small cell lung cancer patients [33]. Furthermore, CD103 is absent from other immune cell populations in the tumor microenvironment, providing excellent cell specificity. Taken together, these studies suggest that the presence of CD103 is a potential biomarker determining T cell infiltration in the tumor microenvironment and, thus, predicting the efficacy of immune checkpoint inhibitors. Recently, [34] two ^89^Zr-anti-human CD103 tracers were preclinically tested in a preclinical setting and high target-to-background ratios, high target site selectivity, and a high sensitivity in human CD103-positive xenografts were found, which offers potential for clinical translation.

Next to these special tissue-resident memory T cells (T_RM_), many other cell surface markers or functional cytokines which are identified in the tumor microenvironment are also investigated as tracers to predict the effects of the immune checkpoint inhibitors.

#### Current immune-targeted PET tracers

As shown in Table 2, PD-L1 remains the primary target for the development of clinical ImmunoPET imaging agents. However, as we discussed at the beginning, PD-1/PD-L1 does not predict response to ICI therapy in some patients, so other targets are being explored. For example, ImmunoPET tracers which target other immune checkpoint molecules such as CTLA-4 and LAG-3 are also investigated in clinical trials. The T cell immunoreceptor (TIGIT) is an inhibitory receptor expressed on T cells and natural killer cells. As an alternative target for checkpoint blockade to the current PD-1/CTLA-4 strategy, antibody-based TIGIT imaging radionuclides were developed and evaluated in vivo in mouse xenograft and synthetic tumor models [35].

**Table 2:** FDA-registered clinical trials of ^89^Zr-labeled immune-targeted PET for cancer applications

ClinicalTrials.gov ID	Target	Phase	Treatment	Sponsor
NCT02453984	PD-L1	Not Applicable	ImmunoPET Imaging With ^89^Zr-MPDL3280A in Patients With Locally Advanced or Metastatic Solid Tumors Prior to and During MPDL3280A Treatment	University Medical Center Groningen
NCT03514719	PD-L1	Phase 1	PD-L1 Imaging in Non Small Cell Lung Cancer’ (PINNACLE)	Radboud University Medical Center
NCT04006522	PD-L1	Phase 2	An Exploratory Study of ^89^Zr-DFO-Atezolizumab ImmunoPET/CT in Patients With Locally Advanced or Metastatic Renal Cell Carcinoma	University of Texas Southwestern Medical Center
NCT04977128	PD-L1	Not Applicable	Safety Study of ^89^Zr-labeled KN035 PET Imaging in Patients With PD-L1 Positive Solid Tumors	Wuxi No. 4 People’s Hospital
NCT03638804	PD-L1	Not Applicable	^89^Zr-labeled KN035 PET Imaging in Patients With PD-L1 positive Advanced Solid Tumors	The First Affiliated Hospital of Soochow University
NCT05742269	PD-L1	Not Applicable	Molecular PD-L1 PET/CT Imaging With ^89^Zr-atezolizumab to Monitor Immune Responses in Metastatic Triple Negative Breast Cancer	Karolinska University Hospital
NCT03850028	PD-L1	Not Applicable	Molecular Imaging of Zirconium-^89^-labeled Atezolizumab as a Tool to Investigate Atezolizumab Biodistribution in High-risk Diffuse Large B-cell Lymphoma	University Medical Center Groningen
NCT05404048	PD-L1	Phase 2	Programmed Death Ligand 1 (PD-L1)-PET Imaging in Patients With (Diffuse) Large B-cell Lymphoma Who Are Treated With CD19-directed CAR T-cell Therapy: a Pilot Study	University Medical Center Groningen
NCT05638334	PD-L1 x 4-1BB	Phase 1	An Open Label, Multicentre, Positron Emission Tomography (PET) Imaging Study Using Zirconium-^89^ to Investigate the Biodistribution and Tumour Uptake of a PD-L1 × 4-1BB Bispecific Antibody (S095012) in Patients With Advanced Solid Tumours	Institut de Recherches Internationales Servier
NCT02760225	PD-1	Not Applicable	^89^Zr-pembrolizumab-PET Imaging in Patients With Locally Advanced or Metastatic Melanoma or Non-small Cell Lung Cancer	University Medical Center Groningen
NCT03065764	PD-1	Phase 2	^89^Zr-labeled Pembrolizumab in Patients With Non-small-cell Lung Cancer	Amsterdam UMC, location VUmc
NCT05068102	PD-1 and SIRPα	Phase 1	An Open Label Phase I PET Imaging Study to Investigate the Bio-distribution and Tumor Uptake of [^89^Zr]Zr-BI 765063 and [^89^Zr]Zr-BI 770371 in Patients With Head and Neck Squamous Cell Carcinoma, Non-small Cell Lung Cancer or Melanoma Who Are Treated With Ezabenlimab	Boehringer Ingelheim
NCT04706715	LAG-3	Phase 1Phase 2	ImmunoPET Imaging With ^89^Zr-DFO-REGN3767 in Patients With Advanced Solid Cancer Prior to and During Treatment With Cemiplimab With or Without Platinum-based Chemotherapy	University Medical Center Groningen
NCT04566978	LAG-3	Early Phase 1	A Pilot Study of ^89^Zr-DFO-REGN3767 Anti LAG-3 Antibody Positron Emission Tomography in Patients With Relapsed/Refractory DLBCL	Memorial Sloan Kettering Cancer Center
NCT03313323	CTLA-4	Phase 2	Uptake and Biodistribution of ^89^Zirconium-labeled Ipilimumab in Ipilimumab Treated Patients With Metastatic Melanoma	Amsterdam UMC, location VUmc
NCT04029181	CD8	Phase 1 Phase 2	ImmunoPET Imaging With ZED88082A in Patients Before and During Treatment With 1) MPDL3280A or 2) PD-1 Antibody Plus or Minus Ipilimumab	University Medical Center Groningen
NCT05259709	CD8	Phase 1	A First-in-Human Study of ^89^Zr-DFO-REGN5054 (Anti-CD8) Positron Emission Tomography in Patients With Solid Malignancies Treated With Cemiplimab	Regeneron Pharmaceuticals
NCT05371132	CD8	Phase 1	Pilot Phase I Study to Evaluate CD8 PET Imaging as a Marker of Immune Response to Stereotactic Body Radiation Therapy (ELIXR)	City of Hope Medical Center
NCT05289193	CD8	Phase 2	CD8 + Cell Imaging During Neoadjuvant ImmunoTherapy (The C-IT Neo Trial)	Memorial Sloan Kettering Cancer Center
NCT05013099	CD8	Phase 2	A Phase IIB, Open Label, Study of Zirconium Zr 89 Crefmirlimab Berdoxam PET/CT in Subjects With Advanced or Metastatic Malignancies, Scheduled to Receive Immunotherapy (IOT) as a Single Agent or Combination, to Predict Response to Therapy	ImaginAb, Inc
NCT03853187	CD8	Phase 2	Imaging Tumor-infiltrating CD8 + T-cells in Non-small Cell Lung Cancer Upon Neo-adjuvant Treatment With Durvalumab (MEDI4736)	Radboud University Medical Center
NCT04955262	CD8	Phase 1	A Phase 1b, Open Label, Multicenter Study of Positron Emission Tomography With Computed Tomography (PET/CT) Using ^89^Zr Df-IAB22M2C (CD8 PET/CT Tracer) in Patients With Metastatic Melanoma Receiving Bempegaldesleukin (NKTR-214) and Nivolumab	Nektar Therapeutics
NCT03533283	CD8	Phase 1 Phase 2	An Open-Label, Multi-Center, Phase IB/II Study of Glofitamab and Atezolizumab or Polatuzumab Vedotin (Plus a Single Pre-Treatment Dose of Obinutuzumab) in Adult Patients With Relapsed/Refractory B-Cell Non-Hodgkin’s Lymphoma	Hoffmann-La Roche
NCT02760199	CD3	Phase 1	^89^Zr-AMG211 PET Imaging in Patients With Relapsed/Refractory Gastrointestinal Adenocarcinoma Before and During Treatment With AMG 211	University Medical Center Groningen

Keywords for searching: Cancer, Immune, ^89^Zr PET, exclude the target expressed on tumor; database: Clinicaltrials.Gov; search date: 2023-10-02

The most potent effectors of the antitumor immune response are CD8^+^ cytotoxic T cells. Therefore, CD8^+^ T cell imaging is currently considered to be the most promising tool for the early identification of immune surveillance function [36]. As shown in Table 2, a variety of clinical programs targeting CD8 are currently underway. However, the presence of CD8^+^ TILs in tumor tissue does not mean that these TILs are functional. A prominent feature of immune escape is T cell depletion, so tracers reflecting the cytotoxic effect of cytotoxic T cells (CTL) may provide additional information for further understanding the immune response. One approach that can show this effect is a tracer targeting granzyme B. Granzyme B is secreted by CD8^+^ T cells and natural killer cells involved in the T cell-mediated tumor cell death process. Such granzyme B tracer was tested in multiple animal models for its potential imaging capabilities [37].

Aside from T cells, which play a pivotal role in combatting tumor cells, natural killer (NK) cells contribute significantly to the immune system’s response against tumors. NK cells, classified as innate lymphoid cells, offer a distinct approach in their interactions with tumor cells compared to T cells. This unique aspect of NK cells is being explored as a potential avenue for treating individuals who do not respond to current immunotherapies [38, 39]. Presently, numerous preclinical studies are underway to track NK cells using the ^89^Zr-oxine in vivo cell labeling method [40–42]. Except for direct cell labeling, NK cell activation receptor NKp30 [43] and CD69 [44], which is an early activation marker expressed on a variety of activated immune cells including NK cells, are also developed into ImmunoPET markers and preclinical tested in the mice model to monitor immunotherapy-induced immune activation.

### ImmunoPET plays a predominant role in detecting the response of immune checkpoint inhibitors

Positron emission tomography (PET) is a molecular imaging technique that allows repetitive, noninvasive clinical assessment of tumor characteristics, such as the expression of hormones and tumor cell metabolism [45–47]. PET is characterized by a high spatial resolution, sensitivity, and the possibility to quantify the imaging signal obtained by administering the appropriate PET-tracer [48]. Compared to biopsy-based techniques, PET could provide a noninvasive, real-time dynamic, whole-body surveillance of certain biomarkers.

Immuno Positron Emission Tomography (ImmunoPET) is a pioneering molecular imaging technique that takes advantage of the superior targeting accuracy of positron emission tomography radiolabeled monoclonal antibodies (mAb), as well as the inherent sensitivity of positron emission tomography imaging. Compared to the small molecule PET tracers, the specificity of the antibody improves tumor detection and provides phenotypic information related to primary and metastatic lesions. Developed by Meijs et al. in [49], the first ^89^Zr-labeled anti-EpCam antibody 323/A3 was successfully applied to visualize human OVCAR-3 xenografts in immune-deficient mice. Since then, many ^89^Zr-labeled antibodies have been developed and broadly applied in cancer imaging [50, 51]. As one review summarized recently [52], PD-L1 is still the predominant target among current ImmunoPET tracer development that is addressed in nearly half (45%, 48 tracers) of all published tracers, followed by PD-1 (10%) and CD8 (9%). Also, as we discussed above, due to the limitations of the PD-L1 targets, various newly discovered tumor-infiltrating targets, such as CD103, capable of directly indicating the immune response have been developed into ImmunoPET tracers to help predict responses to immune checkpoint inhibitors.

Although ImmunoPET gradually plays an increasingly important role in monitoring cancer immune therapy, current ImmunoPET tracers are still limited to specific clinical indications or research purposes. Other issues need to be further investigated, such as the lack of target specificity of the cell surface markers (several lymphocyte lines share many cell surface antigens) and whether the sensitivity of current PET imaging can identify that cell surface antigen. Further large multicenter randomized trials are needed to bring these ImmunoPET tracers into clinical applications.

The conventional biopsy-based IHC is the most widely applied technique to identify specific biomarkers. However, due to the constantly changing expression of the immune target with disease progression and therapy, it is not feasible to visualize the dynamic changes of such targets in vivo through such a biopsy-based invasive method [53]. Furthermore, because the biopsy samples can only be taken from several single lesions, they will not represent the full image of the disease. Compared to IHC, PET imaging with radionuclide-labeled molecules has the advantage of providing a full-version and dynamic picture of the expression of markers in vivo. Both primary and metastatic tumors can be evaluated in a noninvasive manner [54, 55].

Fludeoxyglucose (FDG)-PET has been applied to predict and assess the prognostic effect of immunotherapy. However, the results are not consistent with the clinical outcome of immunotherapy [56, 57]. The increased uptake of [^18^F]FDG is caused by the enhanced metabolic activity of tumor tissue. It does not directly reflect the characteristics of the tumor microenvironment (TME) composition, nor does it identify the phase changes in TME composition during immunotherapy [58].

In conclusion, due to the dynamic expression and heterogeneity of immunological targets, the current assessment methods, such as IHC and fluorodeoxyglucose-positron emission tomography (FDG-PET), are insufficient to evaluate immune therapy’s effectiveness during the preclinical phase and in clinical applications.

Although this paper focuses on the application of ImmunoPET tracers, we recognize that small molecule-based tracers still play a dominant role in the field. Compared to antibodies, small molecules have the potential to be a more accessible and cheaper option due to the lower production costs. They are often easier to handle, as they are relatively stable to pH and heat. The most attractive aspect of small molecule tracer development is that published small molecules that were toxic or ineffective in preclinical or clinical studies can be repurposed as PET tracers during tracer development. For example, a molecule can effectively bind to a specific target but lacks therapeutic efficacy as a drug. However, when utilized as a tracer, it retains its ability to efficiently locate the target without interfering with the target’s function. In the other scenario, when administered at high therapeutic doses, a molecule that may induce adverse side effects or toxicity in the body still demonstrates the capacity to accurately target the desired site without any undesirable effects when formulated as a tracer for use at lower, non-therapeutic doses. This benefit has indeed attracted the development of small molecule tracers targeting PD-L1 [59, 60]. However, the feasibility of this approach for other immune system targets remains to be explored. For example, no small molecule conjugates of CD3 and CD8 have been published for apparent reasons of lack of therapeutic purpose. Whether it is an antagonist or an agonist, it may disrupt the balance of the immune system and affect the immune response. Due to the cost, developing and validating small molecule tracers from scratch may offer fewer advantages than published antibody fragments or minibodies.

Except for PET imaging, there are four other main imaging techniques currently used for tumor diagnosis: X-rays (both plain and computed tomography or CT), ultrasound (US), magnetic resonance imaging (MRI), and optical imaging (OI) [61]. CT, US, and MRI are anatomical imaging methods that lack specificity and focus primarily on demonstrating morphological or density changes [62]. Such imaging methods may even provide misleading information during treatment with immune checkpoint inhibitors due to the "pseudoprogression" that occurs during immunotherapy, where the tumor size initially increases or remains stable and eventually regresses [63].

Fluorescence imaging as a representative of the optical imaging is also explored during immune checkpoint inhibitor therapy to evaluate the therapeutic response. Like PET imaging, fluorescence imaging is a whole-body, noninvasive molecular imaging technique. Compared to ImmunoPET, the cost of fluorescence imaging is relatively low [64]. Various fluorescent probes are tested in preclinical animal models that target T cells or immune regulators, including CD8 [65], CD25 [66], and PD-L1 [67]. However, a few more puzzles still need to be solved before it finally reaches the patients. Such as that the fluorescence signals are often limited by their ability to penetrate tissues, and the endogenous fluorescence from tissue may lead to high background noise. Although compared to others, the near-infrared fluorescence (NIR) imaging which possess the low auto autofluorescence, deep tissue penetration and minimal light scattering features gained a lot of success in last decades by assisting surgeons in identify the right tumor tissue during the surgery [67, 68]. Compared to the PET image, the penetration of this type of imaging is still very limited. Its penetration depth can only reach the epidermis for a few millimeters to a few centimeters, so at present, optical imaging is mainly used in the fundamental research of small animal models [69]. Moreover, with the current novel imaging agents that have been developed as fluorescent probes, the agents’ immunogenicity must be tested concerning immune surveillance. Other safety issues, for example, toxicity and biocompatibility, must also be thoroughly examined before they can reach the patients [70].

## Conclusions

To address the lack of reliable biomarkers in cancer immunotherapy and the inadequacy of current screening methods for these biomarkers, we proposed CD103 ImmunoPET of tissue-resident memory T cell as a potential method for determining TILs load and response to ICIs. Compared to the current biomarkers, such as CD3 CD8, CD103 is a more specific biomarker to a small subgroup of T cells that increases during a successful immune checkpoint inhibitor therapy. However, CD3 and CD8 are more general markers of T cells. Furthermore, tumor-infiltrating lymphocytes represented by CD103 provide a more intuitive picture of the immune response to treatment with ICIs than the widely used target PD-L1.

Among all imaging techniques, ImmunoPET provides valuable information on full-version and dynamic picture of the expression of markers in a noninvasive manner, so it holds tremendous potential in predicting immune response in cancer immunotherapy. However, we should also not neglected the other techniques. In contrast to ImmunoPET which could provide the full-version dynamic changes of the specific markers in a noninvasive way, IHC can provide detailed information about the molecular characteristics of a selected tumor or tissue sample. Ultimately, they provide complementary information to each other.

For the anatomical imaging methods, with improvements in imaging analysis methods, analytical methods such as radiomics [71] can extract a large number of quantitative features from images, which can then be analyzed using advanced computational techniques to gain insight into disease diagnosis, prognosis, and prediction of treatment response. Although this method has not yet been widely applied to predict tumor immune responses due to concerns and changelings in repeatability, reproducibility, and transferability of radiomics features, it will be promising in the future with further development of computer technology [72].

The signal penetration depth and autofluorescence of fluorescence imaging limit its use in most solid tumors. Near-infrared fluorescence imaging, with its low autofluorescence, deep tissue penetration, and low light scattering, has helped surgeons to correctly identify tumor tissues during surgery over the past decade. Due to the tremendous benefits of NIRF imaging in surgery, there is now also research dedicated to developing hybrid tracers that allow for preoperative or postoperative nuclear imaging and intraoperative near-infrared fluorescence (NIRF) imaging, which can aid in accurate preoperative surgical planning and real-time intraoperative tumor detection [70, 73].

In the era of precision medicine and molecularly targeted therapies, the need for targeted imaging has inevitably become a mainstream trend. ImmunoPET holds great promise with its inherent advantages. However, it is not a substitute for other imaging techniques and tests, and the combination of multiple diagnostic methods for different diagnostic and therapeutic purposes in future clinical practice will ultimately provide us with more comprehensive information on the treatment of cancer patients.

## Data Availability

All data relevant to the study are included in the article or uploaded as online supplemental information.

## Abbreviations

ICIs: Immune checkpoint inhibitors
PD-1: Programmed cell death protein
PD-L1: Programmed cell death ligand 1
CTLA-4: Cytotoxic T-lymphocyte-associated antigen 4
MSI-H: High microsatellite instability
dMMR: DNA mismatch repair deficiency
ORR: Objective response rate
IHC: Immunohistochemistry
TMB: Tumor mutational burden
NSCLC: Non-small cell lung cancer
PFS: Progression-free survival
OS: Overall survival
CLL: Chronic lymphocytic leukemia
RT: Richter transformation
DCB: Durable clinical benefit
LDH: Lactate dehydrogenase
TILs: Tumor-infiltrating lymphocytes
TME: Tumor microenvironment
TRM: Tissue-resident memory T cells
PET: Positron emission tomography
